# Effect of Lifestyle Interventions on Gestational Weight Gain Among Pregnant Women with Overweight: A Systematic Review and Meta-Analysis of Randomized Controlled Trials

**DOI:** 10.3390/nu18142258

**Published:** 2026-07-10

**Authors:** Phunthip Chomkhuntod Setthathanapokin, Phatcharaphon Whaikid, Noppawan Piaseu

**Affiliations:** 1 Doctor of Philosophy Program in Nursing Science (International Program), Faculty of Medicine Ramathibodi Hospital, Faculty of Nursing, Mahidol University, Bangkok 10400, Thailand; phunthipchomkhuntod.set@student.mahidol.ac.th; 2 Faculty of Nursing, Huachiew Chalermprakiet University, Samut Prakan 10540, Thailand; phatcharaporn.wha@hcu.ac.th; 3 Ramathibodi School of Nursing, Faculty of Medicine Ramathibodi Hospital, Mahidol University, Bangkok 10400, Thailand; 4 Center for Health Promotion and Well-Being, Faculty of Medicine Ramathibodi Hospital, Mahidol University, Bangkok 10400, Thailand; 5 JBI Evidence-Based Healthcare Ramathibodi School of Nursing Center, University of Adelaide, North Adelaide, SA 5006, Australia

**Keywords:** gestational weight gain, overweight, pregnancy, lifestyle intervention, randomized controlled trial, systematic review, meta-analysis

## Abstract

**Background/Objectives:** Excessive gestational weight gain (GWG) among pregnant women with overweight is associated with adverse maternal and neonatal outcomes. Previous reviews have often combined women with overweight and obesity, limiting overweight-specific evidence. This systematic review and meta-analysis evaluated the effects of lifestyle interventions on GWG among pregnant women with pre-pregnancy overweight. **Methods:** PubMed, Embase, Scopus, and CINAHL were searched for randomized controlled trials (RCTs) published between January 2015 and December 2025. Eligible studies included pregnant women aged ≥ 18 years with pre-pregnancy overweight who received lifestyle interventions incorporating dietary and physical activity (PA) components compared with usual antenatal care. Risk of bias was assessed using the Joanna Briggs Institute (JBI) Critical Appraisal Checklist for RCTs. Random-effects meta-analysis was performed to calculate pooled mean differences (MDs) with 95% confidence intervals (CIs). **Results:** Seventeen reports representing 15 unique RCTs involving 6256 participants were included in the systematic review, of whom 2216 were included in overweight subgroup analyses. Twelve studies contributing data from 1728 women with overweight were included in the meta-analysis. Common support strategies included behavioral support through goal setting and self-monitoring, as well as digital or remote follow-up. Lifestyle interventions were associated with a significant reduction in total GWG compared with usual antenatal care (MD = −1.44 kg; 95% CI: −2.34 to −0.55; *p* < 0.05). Substantial heterogeneity was observed across studies (*I*^2^ = 76%). Limitations included substantial heterogeneity in intervention characteristics, limited reporting of blinding procedures, and the relatively small number of studies available for subgroup analyses. **Conclusions:** Lifestyle interventions incorporating dietary and PA components were associated with significant reductions in GWG among pregnant women with overweight. These findings support the integration of lifestyle interventions into antenatal care to promote appropriate GWG. Further research is needed to identify the most effective intervention components and optimize intervention implementation in this population. **Registration:** PROSPERO CRD42023473693.

## 1. Introduction

Overweight among pregnant women is an increasing global public health concern, with a continuous upward trend observed across regions. The World Health Organization (WHO) defines overweight as a body mass index (BMI) of 25.0–29.9 kg/m^2^, with lower cut-off points applied in Asian populations. Approximately 43.8% of pregnant women worldwide are affected by overweight or obesity, with increasing prevalence over time [1]. Population-level evidence also indicates that excess body weight is common among women before pregnancy, suggesting that an increasing number of women may enter pregnancy with pre-pregnancy overweight or obesity [2]. This trend has important implications for excessive gestational weight gain (GWG) and related maternal and neonatal outcomes.

Pre-pregnancy overweight is associated with an increased risk of adverse maternal and neonatal outcomes. A meta-analysis including more than 20 million pregnant women reported that women with overweight had higher risks of gestational diabetes mellitus, hypertensive disorders, cesarean delivery, and postpartum complications compared with women with normal weight. In addition, maternal overweight was associated with adverse neonatal outcomes, including large for gestational age, macrosomia, preterm birth, and neonatal intensive care unit admission [3]. These findings indicate that overweight is a significant risk factor for pregnancy-related complications. Therefore, appropriate management of GWG is essential to reduce both maternal and neonatal risks.

Appropriate GWG is a key strategy to reduce adverse pregnancy outcomes among women with overweight. GWG is a normal physiological process that supports fetal growth and maternal adaptations during pregnancy. However, both inadequate and excessive GWG are associated with adverse maternal and neonatal outcomes. The Institute of Medicine (IOM) recommends a total GWG of 7.0–11.5 kg for women with pre-pregnancy overweight. This recommendation corresponds to an average weight gain of approximately 0.23–0.33 kg/week during the second and third trimesters. Excessive GWG is defined as total weight gain exceeding the recommended range according to pre-pregnancy BMI. Among women with pre-pregnancy overweight, this refers to a total GWG greater than 11.5 kg [4]. However, a substantial proportion of pregnant women with overweight exceed these recommendations, including evidence from Thailand showing a high prevalence of excessive GWG [5]. Lifestyle interventions during pregnancy, particularly those targeting dietary behaviors and physical activity (PA), are widely recommended to support appropriate GWG. Such interventions may include dietary interventions, physical activity interventions, behavioral support strategies, and technology-assisted approaches designed to promote healthy lifestyle behaviors during pregnancy. Nevertheless, the effectiveness of these interventions remains inconsistent, possibly due to variations in intervention components, intensity, and delivery methods.

Despite the increasing number of systematic reviews on lifestyle interventions during pregnancy, the existing evidence remains inconsistent. Previous reviews have shown that lifestyle interventions may reduce GWG; however, the magnitude of reduction is small and not consistently associated with improved maternal or neonatal outcomes [6]. Other systematic reviews and meta-analyses have reported heterogeneous findings, with variations in intervention components, intensity, and study populations limiting the comparability of results [7]. Although some evidence suggests that dietary and PA interventions can contribute to GWG restriction, the optimal intervention strategy and its overall effectiveness remain unclear [8]. More recent analyses also highlight variability in intervention effects and the limited strength of available evidence, further indicating the need for more focused evaluation [9]. Importantly, most existing reviews have evaluated combined populations of overweight and obese pregnant women, which may limit the applicability of findings to women with overweight alone. Therefore, this systematic review and meta-analysis aimed to evaluate the effectiveness of lifestyle interventions, compared with usual care, on GWG among pregnant women with pre-pregnancy overweight, based on evidence from randomized controlled trials (RCTs).

## 2. Materials and Methods

This systematic review and meta-analysis was conducted and reported in accordance with the Preferred Reporting Items for Systematic Reviews and Meta-Analyses (PRISMA) 2020 statement [10]. The study protocol was prospectively registered in the International Prospective Register of Systematic Reviews (PROSPERO; registration number CRD42023473693) [11]. The review was performed based on predefined eligibility criteria, search strategies, and data synthesis methods.

### 2.1. Eligibility Criteria

The eligibility criteria were defined according to the population–intervention–comparator–outcome–study design (PICOS) framework—population: pregnant women aged ≥ 18 years with pre-pregnancy overweight (BMI 25.0–29.9 kg/m^2^); studies including mixed BMI categories were eligible if data for the overweight subgroup could be extracted separately; intervention: lifestyle interventions delivered during pregnancy, defined as multicomponent interventions that included both dietary behavior and physical activity components, with the aim of promoting healthy lifestyle behaviors and gestational weight management; comparator: usual antenatal care; outcome: GWG; study design: RCTs. Excluded were non-randomized studies, quasi-experimental studies, observational studies, non-controlled trials, pilot and feasibility randomized trials, conference abstracts, study protocols, reviews, animal studies, and articles not published in English.

### 2.2. Information Sources and Search Strategy

A systematic literature search was conducted in PubMed, Embase, Scopus, and CINAHL to identify relevant studies published between January 2015 and December 2025. The publication period was predefined to capture evidence reflecting current clinical practice in gestational weight management among pregnant women with overweight. The search strategy was developed based on key concepts related to pregnancy, overweight, lifestyle interventions, and GWG, using a combination of keywords and controlled vocabulary combined with Boolean operators (AND, OR). The full search strategies for all databases are provided in the Supplementary Materials (see Supplementary Table S1).

### 2.3. Outcome Measures

The primary outcome of this review was total GWG among pregnant women with overweight. Additional outcomes included GWG rate (kg/week) and excessive GWG according to the IOM recommendations, where reported. For studies reporting GWG outcomes at multiple assessment points during pregnancy and postpartum follow-up, data reflecting total GWG or the latest pregnancy assessment before delivery from overweight subgroup analyses were extracted for analysis. Studies providing sufficient quantitative outcome data were included in the meta-analysis, whereas studies reporting outcomes in other formats or without sufficient quantitative data were included in the qualitative synthesis. For trials reported in multiple publications, outcome data were extracted from the report providing the most complete and relevant information to avoid duplicate inclusion.

### 2.4. Study Selection and Data Extraction

All identified records were imported into EndNote 21 (Clarivate Analytics, Philadelphia, PA, USA), and duplicate records were removed. Two independent reviewers (P.C.S. and N.P.) independently screened the titles and abstracts of all identified records according to the predefined inclusion and exclusion criteria. Potentially eligible reports were retrieved for full-text assessment. Full-text reports were independently assessed for eligibility by the same reviewers. Any disagreements during the study selection process were resolved through discussion or consultation with a third reviewer (P.W.) to ensure consistency with the predefined eligibility criteria. The study selection process was documented and reported using the PRISMA 2020 flow diagram.

Data extraction was conducted independently by two reviewers (P.C.S. and N.P.) using a standardized data extraction form developed for this review to ensure consistency and accuracy in data collection. Any disagreements during the data extraction process were resolved through discussion or consultation with a third reviewer (P.W.). No contact with study investigators was required to obtain or confirm study data. Data extracted for study characteristics included (1) author and year of publication, (2) country, (3) participant characteristics, including maternal age and gestational age at eligibility and baseline, BMI category targeted in the studies, total sample size, and overweight subgroup sample size, (4) intervention summary, and (5) GWG outcomes, including total GWG, GWG rate, and excessive GWG. Data extracted for intervention characteristics included (1) dietary procedures, (2) physical activity procedures, (3) modes of delivery, (4) duration of program, and (5) core components. When relevant information was unclear or incompletely reported, Supplementary Materials and related publications from the same trial were reviewed for additional details. Studies without sufficient quantitative outcome data were included in the qualitative synthesis only.

When multiple publications originated from the same trial, they were identified using study characteristics and participant information and were treated as a single study to avoid double counting. Data were extracted from the publication(s) providing the most complete and relevant information for each variable.

### 2.5. Risk of Bias Assessment

The methodological quality of the included studies was assessed using the Joanna Briggs Institute (JBI) Critical Appraisal Checklist for RCTs [12], which consists of 13 items evaluating the risk of bias in key methodological domains. These domains include randomization procedures, allocation concealment, baseline comparability of groups, blinding of participants, intervention providers, and outcome assessors, similarity of treatment between groups, completeness of follow-up, intention-to-treat analysis, consistency and reliability of outcome measurement, appropriateness of statistical analysis, and suitability of the trial design.

Two reviewers (P.C.S. and N.P.) independently evaluated each study using the checklist. Each item was rated as “yes,” “no,” “unclear,” or “not applicable” according to JBI guidance. Studies were not excluded based on quality assessment. Disagreements between reviewers were resolved through discussion or consultation with a third reviewer (P.W.).

### 2.6. Data Synthesis and Statistical Analysis

Study findings were synthesized and presented in tables summarizing study characteristics, participant characteristics, intervention characteristics, and GWG outcomes. Meta-analysis results were visually presented using forest plots.

Where studies were sufficiently comparable in terms of participant characteristics, lifestyle intervention components, and outcome reporting, and provided sufficient quantitative data for pooling, a meta-analysis was performed using JBI SUMARI (Joanna Briggs Institute, Adelaide, Australia) [13]. For continuous outcomes, effect estimates were calculated as mean differences (MDs) with 95% confidence intervals (CIs) using a random-effects model. Statistical heterogeneity was assessed using the *I*^2^ statistic and chi-square test, with a *p*-value < 0.1 indicating significant heterogeneity. *I*^2^ values of <25.0%, 25.0–49.9%, 50.0–74.9%, and ≥75.0% were interpreted as low, moderate, substantial, and considerable heterogeneity, respectively.

Where sufficient data were not available for quantitative synthesis, studies were included in the qualitative synthesis. Sensitivity analysis was performed to assess the robustness of the pooled estimates by sequentially omitting individual studies. A *p*-value < 0.05 was considered statistically significant. Publication bias was assessed by visual inspection of a funnel plot because more than 10 studies were included in the meta-analysis.

## 3. Results

### 3.1. Selection of Included Studies

A total of 1477 records were identified through database searching (PubMed, *n* = 337; Scopus, *n* = 499; Embase, *n* = 343; and CINAHL, *n* = 298). After removing 658 duplicate records, 819 records remained for title and abstract screening. Of these, 758 records were excluded, and 61 reports were sought for full-text retrieval. One report could not be retrieved, resulting in 60 reports assessed for eligibility.

Of the 60 reports, 43 were excluded for the following reasons: no separate data for the overweight subgroup (*n* = 29), interventions not consistent with the eligibility criteria (*n* = 9), comparators other than usual care (*n* = 3), and articles not published in English (*n* = 2).

**Figure 1:**
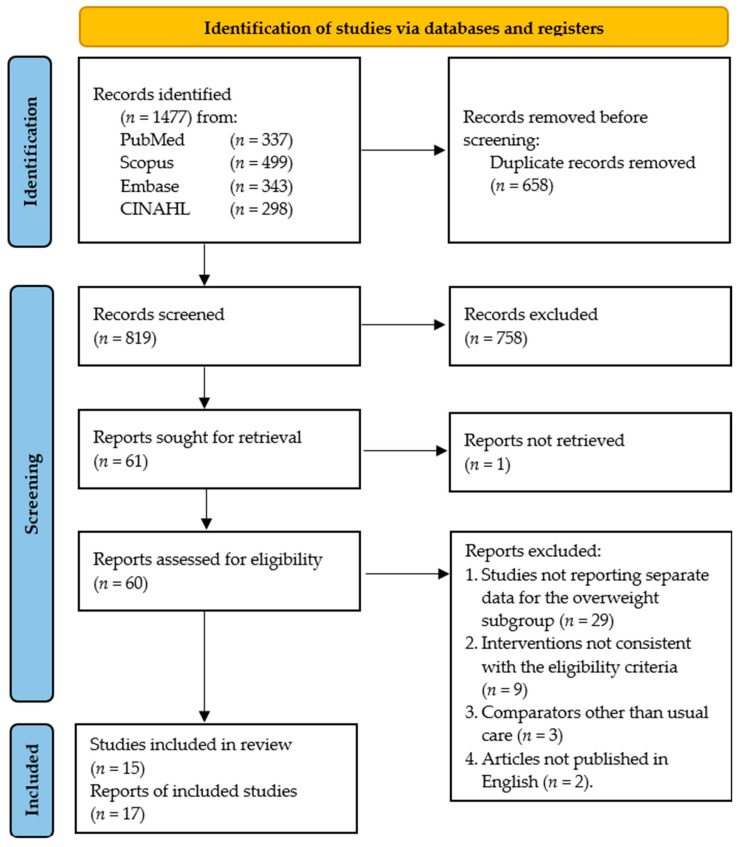
PRISMA 2020 flow diagram of the study selection process.

Finally, 17 reports (publications) representing 15 unique studies (trials) were included in the review after multiple reports from the same trial were collated. Of these, 12 studies provided sufficient data for inclusion in the meta-analysis. The study selection process is presented in Figure 1.

### 3.2. Study Characteristics

The 15 included RCTs were published between 2017 and 2025 [14,15,16,17,18,19,20,21,22,23,24,25,26,27,28,29,30]. Included studies originated from the United States (*n* = 7) [14,16,17,18,20,22,28,29], Germany (*n* = 2) [19,24], Taiwan (*n* = 3 reports from two trials) [26,27,30], and one study each from Norway [15], Iran [21], Canada [23], and Brazil [25].

Across all included studies, 6256 participants were enrolled, with 2216 participants included in overweight subgroup analyses. Mean maternal age ranged from approximately 25 to 36 years, and gestational age at enrollment ranged from approximately 8 to 19 weeks. Six studies included participants with overweight and obesity (OW/OB), six included all BMI categories, and three included only participants with overweight (OW only). Dietary interventions were most commonly classified as Counseling and Monitoring (*n* = 8) [16,17,20,21,22,26,27,28,29,30], followed by Structured Dietary (*n* = 4) [14,18,23,25] and Counseling (*n* = 3) [15,19,24]. Similarly, PA interventions were predominantly classified as Counseling and Monitoring (*n* = 8) [17,18,19,20,22,26,27,28,29,30], followed by Counseling (*n* = 4) [14,16,24,25] and Structured PA (*n* = 3) [15,21,23].

Outcome data in this review were extracted from overweight group analyses. Most studies reported total GWG, while some additionally reported GWG rate and excessive GWG. Overall, intervention groups generally demonstrated lower GWG outcomes compared with control groups. Total GWG ranged from approximately 8.9 to 15.3 kg in intervention groups and from 9.2 to 17.7 kg in control groups. Among studies reporting GWG rate, lower weekly weight gain was generally observed in intervention groups than in control groups [16,20,23,25]. Excessive GWG was also less frequent among participants receiving lifestyle interventions in most studies. The proportion of excessive GWG ranged from 20.0% to 85.0% in intervention groups and from 30.3% to 100% in control groups [16,17,18,19,21,22,24,25,26,27,28,29,30], although the magnitude of the effect varied across studies. Detailed study characteristics are presented in Table 1.

Regarding intervention characteristics, dietary procedures commonly included healthy eating counseling, individualized dietary guidance, calorie or portion control, weight monitoring, and dietary self-monitoring [15,16,17,19,20,21,22,26,27,28,29,30]. Several studies implemented structured dietary approaches, including low-carbohydrate, carbohydrate-controlled, high-protein dairy, and minimally processed food-based diets [14,18,23,25]. Physical activity procedures commonly promoted walking, step-count goals, and achievement of at least 150 min/week of moderate-intensity PA [17,18,19,20,22,26,27,28,29,30], while some studies incorporated supervised or prescribed aerobic, strength, stretching, or walking exercise programs [15,21,23].

Modes of delivery most commonly combined face-to-face and technology-based approaches [14,15,17,18,20,21,22,23,24], followed by technology-only delivery using mobile applications, wearable activity trackers, and SMS communication [26,27,28,29,30], while fewer studies used face-to-face delivery alone [16,19,25]. Intervention duration ranged from 19 to 26 weeks, with most studies lasting 22–24 weeks.

Core components frequently included goal setting, self-monitoring, feedback, reminders, motivational interviewing, social support, coaching, and problem solving [17,18,20,22,23,24,26,27,28,29,30]. Supervised exercise was incorporated in two studies [15,23], whereas incentive- or reward-based strategies were reported in technology-based interventions [26,27,28,29,30]. Detailed intervention characteristics are presented in Table 2, with additional information on intervention materials, providers, and settings provided in Supplementary Table S2.

### 3.3. Meta-Analysis

**Figure 2:**
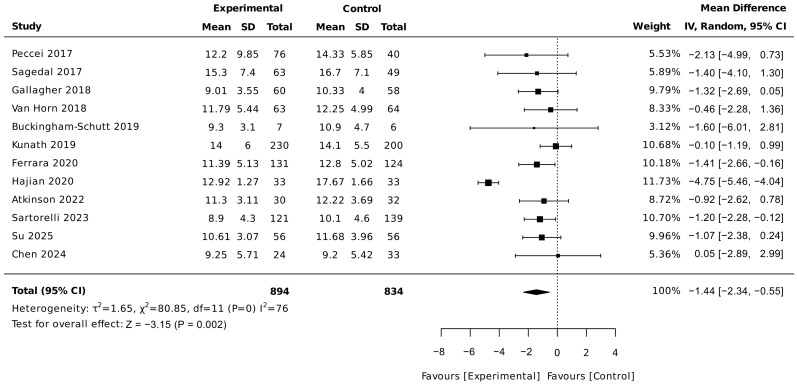
Forest plot of the pooled effects of lifestyle interventions on GWG among pregnant women with overweight [14,15,16,17,18,19,20,21,23,25,27,30].

The pooled analysis included 12 studies with 1728 participants (894 in the intervention group and 834 in the control group). Compared with the control group, the intervention group showed a significant reduction in total GWG (MD = −1.44 kg, 95% CI: −2.34 to −0.55) (Figure 2). Most included studies demonstrated lower GWG in the intervention group compared with the control group, although the magnitude of effect varied across studies. Substantial heterogeneity was observed across studies (*I*^2^ = 76%, *p* < 0.05).

### 3.4. Sensitivity Analyses

Sensitivity analyses using the leave-one-out method demonstrated that no single study altered the direction or statistical significance of the pooled effect estimate. The pooled effect estimates remained statistically significant after sequential exclusion of individual studies, ranging from −0.18 to −0.43, and all analyses favored the intervention group. Detailed results of the sensitivity analysis are provided in Supplementary Figure S1.

### 3.5. Risk of Bias in Studies

The results of the Joanna Briggs Institute (JBI) Critical Appraisal Checklist for RCTs are presented in Table 3. Overall, the included studies demonstrated generally good methodological quality.

All included studies met the criteria for appropriate randomization procedures, complete follow-up or adequate management of attrition, consistent and reliable outcome measurement, appropriate statistical analyses, and suitable trial designs. In addition, most studies reported comparable baseline characteristics between groups, appropriate treatment of participants according to their randomized allocation, and identical management of treatment groups apart from the intervention of interest.

The main methodological concerns were related to allocation concealment and blinding procedures. Adequate allocation concealment was clearly reported in only seven studies, whereas eight studies provided insufficient information. Blinding of outcome assessors was reported in five studies, while eight studies were rated as unclear and two studies did not implement assessor blinding.

Participant blinding and treatment-provider blinding were not reported in any of the included studies. This finding is common in behavioral and exercise-based interventions, where blinding is often impractical. Overall, the risk of bias was considered low to moderate, with limitations primarily related to allocation concealment and blinding procedures.

### 3.6. Publication Bias

**Figure 3:**
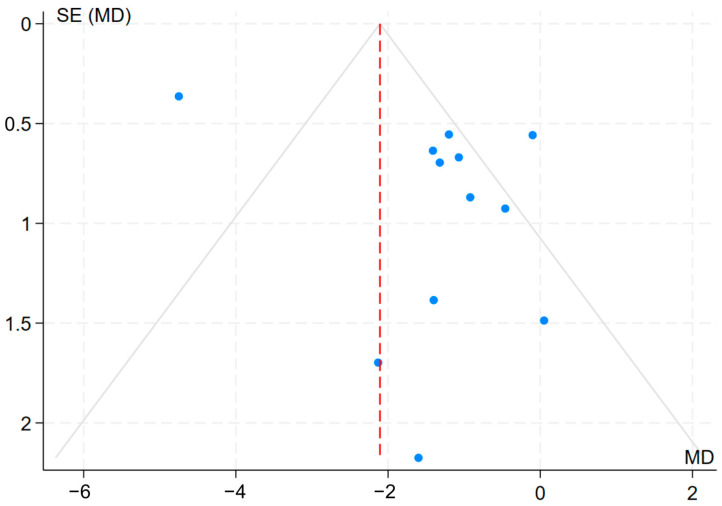
Funnel plot for assessment of publication bias. Blue dots represent individual studies, solid lines indicate the pseudo 95% confidence limits, and the red dashed line indicates the pooled effect estimate. MD, mean difference; SE, standard error.

Publication bias was assessed using a funnel plot generated in Stata Statistical Soft-ware, Release 18 (StataCorp LLC, College Station, TX, USA). As shown in Figure 3, the studies were symmetrically distributed around the pooled effect estimate, indicating no evidence of publication bias.

## 4. Discussion

Our systematic review and meta-analysis demonstrated that lifestyle interventions significantly reduced GWG among pregnant women with overweight (pre-pregnancy BMI 25.0–29.9 kg/m^2^), with a pooled reduction of 1.44 kg compared with usual antenatal care. Although the pooled reduction of 1.44 kg was statistically significant, its clinical significance should be interpreted with caution. The included studies primarily evaluated gestational weight gain rather than maternal or neonatal clinical outcomes; therefore, it remains unclear whether this magnitude of GWG reduction directly translates into improved maternal or neonatal outcomes.

To our knowledge, this is the first systematic review and meta-analysis specifically evaluating the effects of lifestyle interventions on GWG among pregnant women with overweight. Previous systematic reviews and meta-analyses evaluating lifestyle interventions during pregnancy have generally included women across all BMI categories [31,32,33,34] or combined women with overweight and obesity [35,36,37] into a single population. Consequently, evidence regarding the effectiveness of lifestyle interventions specifically among women with overweight has remained limited. This represents an important evidence gap because women with overweight already have an increased risk of adverse maternal and neonatal outcomes compared with women with normal BMI. Moreover, intervention effectiveness differs according to maternal BMI status [38]. Therefore, evaluating interventions within a homogeneous BMI category is important, as women with similar BMI characteristics may respond differently to lifestyle interventions than those in other BMI groups. From a clinical implementation perspective, the included studies suggest that several feasible components could be incorporated into routine antenatal care, particularly in resource-limited settings. These components include brief dietary and physical activity counseling, individualized goal setting, regular weight monitoring, self-monitoring of lifestyle behaviors, and periodic feedback. Low-cost digital or remote follow-up approaches, such as mobile applications, text messaging, or telephone-based support, may also help maintain engagement while reducing the need for frequent face-to-face visits. However, because the included studies varied substantially in intervention components, intensity, duration, and delivery modes, the present review cannot determine which delivery model is most effective.

Dietary interventions are effective in reducing GWG, although their effects may be less consistent among women with overweight or obesity [39,40]. Evidence from pregnancy-related dietary interventions has shown an approximately 23% relative reduction in GWG compared with control groups [41]. The dietary components of these interventions were generally consistent with international nutritional recommendations and emphasized increased consumption of nutrient-dense foods. Common recommendations included increasing fruit and vegetable intake to approximately 2–3 servings per day, consuming 6–8 servings of carbohydrate-rich foods, and consuming 2–3 servings of lean protein sources and low-fat dairy products per day. They also promoted reductions in sugar-sweetened beverages and energy-dense foods [4,42]. In addition, our study incorporated calorie-management strategies, including calorie-controlled meal plans, individualized energy targets, portion control, and regular GWG monitoring. Consistent with current recommendations, additional energy requirements during the second and third trimesters were typically targeted at approximately 340–450 kcal/day, resulting in a total daily energy intake of around 2200–2500 kcal, adjusted according to individual needs [4,42].

Physical activity interventions have been recognized as an important strategy for managing GWG among pregnant women with overweight or obesity [43]. A recent systematic review and meta-analysis reported that PA interventions significantly reduced GWG compared with usual care [44]. Furthermore, dose–response evidence indicates that greater engagement in PA is associated with lower GWG, with approximately 5–15 additional minutes of moderate-to-vigorous physical activity per day associated with an estimated 0.50 kg reduction in GWG among women with overweight or obesity [41]. In the present review, these interventions primarily promoted walking, step-count goals ranging from approximately 8500–10,000 steps per day, and achievement of at least 150 min of moderate-intensity physical activity per week or 30 min per day. These intervention characteristics are consistent with recommendations from the American College of Obstetricians and Gynecologists (ACOG) [45] and the WHO [46], which encourage pregnant women without contraindications to engage in regular moderate-intensity physical activity while minimizing sedentary behavior. The alignment between the interventions identified in this review, existing evidence, and current clinical recommendations supports the incorporation of regular PA into antenatal weight management strategies for pregnant women with overweight or obesity.

Several core components were frequently incorporated across the included interventions, particularly goal setting, self-monitoring, feedback, reminders, motivational interviewing, social support, coaching, and problem-solving strategies. Goal setting, self-monitoring, and feedback were the most commonly reported components. Previous evidence has shown that combined lifestyle interventions incorporating multiple behavior-change components were associated with reductions in GWG [7]. Likewise, interventions incorporating multiple core components have been identified as among the most effective approaches for reducing GWG among pregnant women with overweight or obesity [47]. Their frequent inclusion suggests that these components may be important elements of successful gestational weight management interventions for women with overweight. These components were delivered through repeated contacts over 19–26 weeks using face-to-face, technology-based, or blended approaches, with ongoing monitoring throughout pregnancy. Collectively, these findings suggest that successful gestational weight management interventions for women with overweight require not only dietary and physical activity guidance, but also behavioral support delivered through continued contact and monitoring.

The findings of the present review have important implications for antenatal care practice. Given that lifestyle interventions were associated with significant reductions in GWG among pregnant women with overweight, early identification of women at risk of excessive GWG and timely provision of lifestyle support should be considered as part of routine antenatal care. The interventions included in this review were delivered through a range of modalities, including face-to-face, digital, and blended approaches, and were implemented by various healthcare professionals across clinic- and home-based settings, suggesting that gestational weight management support can be adapted to different healthcare contexts and available resources. Previous evidence also indicates that lifestyle interventions delivered within antenatal care settings may contribute to more appropriate GWG [48]. Qualitative evidence further emphasizes that antenatal weight-management care should be responsive to women’s individual circumstances, priorities, and experiences [49]. Collectively, these findings support the integration of tailored lifestyle interventions into routine antenatal care for pregnant women with overweight.

One strength of our meta-analysis is its focus on pregnant women with overweight, a population frequently combined with women with obesity in previous reviews, thereby addressing an important evidence gap. Furthermore, only RCTs were included, strengthening the validity of the synthesized evidence and reducing the risk of bias associated with non-randomized study designs. Limitations in this systematic review and meta-analysis include the following: First, substantial heterogeneity was observed across the included studies. This may be attributable to variations in lifestyle intervention characteristics, including intervention components, modes of delivery, duration, providers, materials, and implementation settings. Such variability limited the ability to conduct meaningful subgroup analyses and further explore potential sources of heterogeneity. Nevertheless, sensitivity analyses supported the robustness of the overall findings despite the substantial heterogeneity. Second, several included studies had methodological weaknesses identified during critical appraisal, particularly regarding the blinding of intervention providers, blinding of outcome assessors, and consistency of treatment between groups beyond the intervention. These limitations may have introduced bias into the pooled estimates. In particular, the lack of participant and provider blinding may have increased the risk of performance bias, while self-reported physical activity and dietary adherence may have contributed to detection or reporting bias. Nevertheless, the leave-one-out sensitivity analysis provided additional support for the robustness of the overall finding. Although the magnitude of the pooled estimate varied when individual studies were sequentially excluded, the direction of effect and statistical significance were maintained in all analyses. This suggests that the observed benefit of lifestyle interventions was not driven by any single study. However, the substantial heterogeneity across studies (*I*^2^ = 76%) should still be considered when interpreting the clinical meaning of this finding. Finally, only English-language publications were included in this review, which may have resulted in the omission of relevant studies published in other languages and may limit the generalizability of the findings across different cultural and linguistic contexts.

Our systematic review and meta-analysis demonstrated that lifestyle interventions incorporating dietary and physical activity components were associated with significant reductions in GWG among pregnant women with overweight. These findings provide valuable evidence for the design and implementation of tailored interventions targeting the specific needs of this population.

## 5. Conclusions

This systematic review and meta-analysis demonstrated that lifestyle interventions incorporating dietary and physical activity components were associated with significant reductions in GWG among pregnant women with overweight. These findings support the integration of tailored lifestyle intervention components into routine antenatal care to promote appropriate GWG in this population. In resource-limited clinical settings, feasible strategies may include brief dietary and physical activity counseling, individualized goal setting, weight self-monitoring, and low-cost remote follow-up to support continued engagement. Despite substantial variation in intervention characteristics across studies, the overall direction of the findings favored lifestyle interventions; however, the clinical significance of the pooled GWG reduction should be interpreted cautiously. Further high-quality studies are needed to determine whether reductions in GWG translate into meaningful improvements in maternal and neonatal clinical outcomes and to identify the intervention characteristics most strongly associated with effectiveness among pregnant women with overweight.

## Figures and Tables

**Table 1:** Study characteristics of the included studies.

No.	Author (Year)	Country	Participant Characteristics							Intervention Summary	Weight Outcomes		
			Age (Years)		GA (Weeks)		BMI Category	Total *n* (I/C)	OW *n* (I/C)		Total GWG (kg), Mean (SD)	GWG Rate (kg/Week), Mean (SD)	Excess GWG (%)
			Eligibility	Baseline, Mean (SD)	Eligibility	Baseline							
1	Peccei (2017) [14]	USA	18–49	NA	<16	NA	OW/OB	180/92	76/40	StD, CPA	12.20 (9.85) 14.33 (5.85)	NA	NA
2	Sagedal (2017) [15]	Norway	≥18	27.9 (4.2) 28.1 (4.5)	≤20	15.4 (2.6) 15.6 (2.4)	All	267/266	63/49	CD, StPA	15.3 (7.4) 16.7 (7.1)	NA	NA
3	Gallagher (2018) [16]	USA	≥18	33.8 (4.0) 33.8 (4.7)	9–15	14.96 (0.72) 14.82 (0.78)	OW/OB	97/99	60/58	CMD, CPA	9.01 (3.55) 10.33 (4.00)	0.25 (0.10) 0.29 (0.11)	20 38
4	Van Horn (2018) [17]	USA	18–45	33 (4) 34 (4)	<16	13.0 (1.0) 13.0 (1.0)	OW/OB	140/140	63/64	CMD, CMPA	11.79 (5.44) 12.25 (4.99)	NA	79 86
5	Buckingham-Schutt (2019) [18]	USA	18–45	31.6 (4.6) 31.2 (3.6)	8–14	NA	All	23/24	7/6	StD, CMPA	9.3 (3.1) 10.9 (4.8)	NA	28.6 83.4
6	Kunath (2019) [19]	Germany	18–43	30.2 (4.4) 30.4 (4.7)	≤12	8.1 (2.1) 8.4 (2.2)	All	946/939	230/200	CD, CMPA	14.0 (6.0) 14.1 (5.5)	NA	65.2 69.0
7	Ferrara (2020) [20]	USA	≥18	32.4 (4.1) 32.6 (4.3)	8–15	14.2 (1.4) 14.4 (1.3)	OW/OB	199/195	131/124	CMD, CMPA	11.39 (5.13) 12.80 (5.02)	0.28 (0.14) 0.33 (0.13)	NA
8	Hajian (2020) [21]	Iran	18–40	25.94 (4.22) 25.06 (3.43)	16–20	18.73 (1.38) 18.42 (1.30)	OW	33/33	33/33	CMD, StPA	12.92 (1.27) 17.67 (1.66)	NA	84.8 100
9	Liu (2021) [22]	USA	18–44	30.4 (5.1) 29.1 (4.8)	≤16	12.6 (2.3) 12.6 (2.3)	OW/OB	112/105	56/49	CMD, CMPA	NA	NA	AA: 61.2 90.3 W: 67.5 67.2
10	Atkinson (2022) [23]	Canada	NA	31.6 (3.90) 31.3 (4.3)	12–17	13.75 (1.75) 13.60 (1.61)	All	105/112	30/32	StD, StPA	11.30 (3.11) 12.22 (3.69)	0.48 (0.13) 0.53 (0.15)	NA
11	Krebs (2022) [24]	Germany	≥18	31.3 (4.3) 31.3 (4.4)	<12	9.9 (1.9) 9.9 (2.0)	All	744/636	172/132	CD, CPA	13.9 (NA) 15.6 (NA) ^1^	NA	78.2 81.1
12	Sartorelli (2023) [25]	Brazil	≥18	NA	≤15	NA	OW	121/139	121/139	StD, CPA	8.9 (4.3) 10.1 (4.6)	0.43 (0.19) 0.47 (0.21)	62.0 73.4
13	Chen (2023 [26]; 2024 [27])	Taiwan	≥20	32.8 (4.96)	<17	13.91 (NA)	OW/OB	37/43	24/33	CMD, CMPA	9.25 (5.71) 9.20 (5.42) ^2^	NA	29.2 30.3 ^3^
14	Cabre (2025) [28]; Kebbe (2025) [29]	USA	18–40	27.0 (6.0) 28.0 (6.0)	10–16	15.2 (1.6) 15.1 (1.6)	All	163/154	42/37	CMD, CMPA	NA	NA	85 86 ^4^
15	Su (2025) [30]	Taiwan	≥18	35.71 (4.31) 35.82 (4.28)	<20	16.40 (4.11) 15.78 (2.91)	OW	56/56	56/56	CMD, CMPA	10.61 (3.07) 11.68 (3.96)	NA	26.0 47.7

^1^ The between-group difference in total GWG was −1.69 kg (95% CI: −2.65 to −0.74). ^2^ Data were primarily extracted from Chen et al. [27]. ^3^ Information on excessive gestational weight gain (%) was obtained from Chen et al. [26]. ^4^ Overweight subgroup outcomes were extracted from Cabre et al. [28] and were based on participants with available outcome assessments. Abbreviations: AA, African American; All, all BMI categories included; BMI, body mass index; CD, Counseling Dietary; CI, confidence interval; CMD, Counseling and Monitoring Dietary; CMPA, Counseling and Monitoring Physical Activity; CPA, Counseling Physical Activity; GA, gestational age; GWG, gestational weight gain; I/C, intervention/control; NA, not available; OB, obesity; OW, overweight; SD, standard deviation; StD, Structured Dietary; StPA, Structured Physical Activity; USA, United States of America; W, White.

**Table 2:** Lifestyle intervention characteristics of the included studies.

No.	Author (Year)	Dietary Procedures	PA Procedures	Modes of Delivery	Duration of Program	Core Intervention
1	Peccei (2017) [14]	Individualized meal plans, low-carbohydrate diet counseling, label reading, healthy food shopping, and nutrition counseling (StD)	Walking > 30 min/day, pedometer use, exercise review during sessions (CPA)	Face-to-face and technology	24-w, 12 sessions (10–30 min/session)	Self-monitoring, Feedback
2	Sagedal (2017) [15]	Portion size, meal patterns, limiting snacks, increasing water/fruits/vegetables intake (CD)	Group aerobic, strength, and stretching exercises; moderate PA 3 days/week (StPA)	Face-to-face and technology	24-w, 2 diet sessions (20 min/session) and 48 PA sessions (60 min/session)	Supervised exercise, Self-monitoring
3	Gallagher (2018) [16]	Calorie-controlled healthy diet, portion control, meal replacement, healthy food choices, and self-monitoring to achieve appropriate GWG (CMD)	Walking, increased PA, step-count goals (10,000 steps/day) (CPA)	Face-to-face	21-w, 10 sessions	Social support, Behavioral support
4	Van Horn (2018) [17]	Calorie-controlled DASH-based diet promoting fruits, vegetables, whole grains, low-fat dairy, and reduced sugar-sweetened/non-nutrient-dense foods (CMD)	>30 min/day or >10,000 steps/day (CMPA)	Face-to-face and technology	22-w, 9 sessions	Motivational interviewing
5	Buckingham-Schutt (2019) [18]	Carbohydrate-controlled meal plan emphasizing whole grains, lean proteins, and unsaturated fats (StD)	Walking 10,000 steps/day, increased MVPA (CMPA)	Face-to-face and technology	22-w, ≥6 sessions (15–30 min/session)	SDT, MI, Feedback
6	Kunath (2019) [19]	Balanced healthy diet promoting appropriate GWG, healthy nutrient intake, and avoiding alcohol/tobacco and food-borne infections (CD)	≥150 min/week of moderate-intensity PA (CMPA)	Face-to-face	24-w, 3 sessions (30–45 min/session)	Self-monitoring, Feedback
7	Ferrara (2020) [20]	Healthy eating goals focused on portion size, calorie intake, and fat intake (CMD)	150 min/week moderate-to-vigorous PA and reduced sedentary behavior (CMPA)	Face-to-face and technology	24-w, 13 sessions (30 min/session)	Motivational interviewing, Goal setting, Self-monitoring
8	Hajian (2020) [21]	Balanced and flexible diet with correction of nutritional misconceptions and healthy eating guidance during pregnancy (CMD)	Aerobic exercise ≥ 3 times/week, walking 20–30 min/day, and stretching exercises (StPA)	Face-to-face and technology	20-w, sessions (30–45 min/session)	NA
9	Liu (2021) [22]	MyPlate guidance, customized calorie goals, healthy diet high in fruits/vegetables/whole grains and low in saturated/trans fats (CMD)	≥150 min/week moderate-intensity PA (CMPA)	Face-to-face and technology	26-w, ≥11 sessions	Goal setting, Self-monitoring, Feedback, Problem solving, Social support
10	Atkinson (2022) [23]	Individualized high-protein dairy diet, energy requirements calculated individually, nutrition counseling with recipes and meal plans (StD)	Walking 3–4 times/week starting 25 min/session and increasing by 2 min weekly to 40 min/session; goal 10,000 steps/day (StPA)	Face-to-face and technology	26-w, 20 diet sessions and 78 PA sessions (25–40 min/session)	Goal setting, Supervised exercise, Adherence support
11	Krebs (2022) [24]	Brief counseling on diet and healthy lifestyle topics based on recommendations (CD)	PA counseling integrated into prenatal care (CPA)	Face-to-face and technology	24-w, ≥6 sessions (10 min/session)	Motivational interviewing, Goal setting, Reminders, Self-regulation
12	Sartorelli (2023) [25]	Promoting minimally processed foods, increased fruit and vegetable intake, reduced ultra-processed foods and sugar-sweetened beverages (StD)	≥150 min/week PA (CPA)	Face-to-face	19-w, 3 sessions (30 min/session)	NA
13	Chen (2023 [26]; 2024 [27])	GWG goal setting according to IOM recommendations (CMD)	Walking 8500 steps/day (CMPA)	Technology	20-w, 20 contacts (app)	Goal setting, Self-monitoring, Feedback, Reminders, Incentives/rewards
14	Cabre (2025) [28]; Kebbe (2025) [29]	Healthy eating, portion control, healthy recipes using WIC foods, weight monitoring, and personalized dietary feedback (CMD)	≥150 min/week moderate PA, walking 5000–10,000 steps/day, exercise videos, reduction in sedentary time (CMPA)	Technology	24-w, 24 contacts (app)	Goal setting, Self-monitoring; Feedback, Social support, Coaching, Reminders
15	Su (2025) [30]	Guidance to avoid high-sugar/high-fat foods, encouragement of balanced intake from six food groups, customized dietary (CMD)	Exercise plans, ≥30 min moderate PA/day for 3–5 days/week, maintenance or initiation of exercise (CMPA)	Technology	22-w, ≥22 contacts (app)	Goal setting, Self-monitoring, Feedback, Reminders, Coaching, Incentives/rewards, Social support

Abbreviations: app, application; CD, Counseling Dietary; CMD, Counseling and Monitoring Dietary; CMPA, Counseling and Monitoring Physical Activity; CPA, Counseling Physical Activity; DASH, Dietary Approaches to Stop Hypertension; GWG, gestational weight gain; IOM, Institute of Medicine; MI, motivational interviewing; MVPA, moderate-to-vigorous physical activity; NA, not available; PA, physical activity; SDT, self-determination theory; StD, Structured Dietary; StPA, Structured Physical Activity; WIC, Special Supplemental Nutrition Program for Women, Infants, and Children; w, week.

**Table 3:** Risk of bias assessment using the JBI critical appraisal tool.

No	Author (Year)	Q1	Q2	Q3	Q4	Q5	Q6	Q7	Q8	Q9	Q10	Q11	Q12	Q13	Total Score *
1	Peccei (2017) [14]	Y	U	Y	N	N	U	Y	Y	Y	Y	Y	Y	Y	9
2	Sagedal (2017) [15]	Y	Y	Y	N	N	Y	Y	Y	Y	Y	Y	Y	Y	11
3	Gallagher (2018) [16]	Y	Y	Y	N	N	U	Y	Y	Y	Y	Y	Y	Y	10
4	Van Horn (2018) [17]	Y	Y	Y	N	N	Y	Y	Y	Y	Y	Y	Y	Y	11
5	Buckingham-Schutt (2019) [18]	Y	U	Y	N	N	U	Y	Y	Y	Y	Y	Y	Y	9
6	Kunath (2019) [19]	Y	U	Y	N	N	U	Y	Y	U	Y	Y	Y	Y	8
7	Ferrara (2020) [20]	Y	Y	Y	N	N	Y	Y	Y	Y	Y	Y	Y	Y	11
8	Hajian (2020) [21]	Y	U	Y	N	N	N	Y	Y	Y	Y	Y	Y	Y	9
9	Liu (2021) [22]	Y	Y	Y	N	N	Y	Y	Y	Y	Y	Y	Y	Y	11
10	Atkinson (2022) [23]	Y	U	N	N	N	U	Y	Y	Y	Y	Y	Y	Y	8
11	Krebs (2022) [24]	Y	U	Y	N	N	U	N	Y	Y	Y	Y	Y	Y	8
12	Sartorelli (2023) [25]	Y	U	Y	N	N	N	Y	Y	Y	Y	Y	Y	Y	9
13	Chen (2023 [26]; 2024 [27])	Y	U	Y	N	N	U	Y	Y	Y	Y	Y	Y	Y	9
14	Cabre (2025) [28]; Kebbe (2025) [29]	Y	Y	Y	N	N	Y	Y	Y	Y	Y	Y	Y	Y	11
15	Su (2025) [30]	Y	Y	Y	N	N	U	Y	Y	Y	Y	Y	Y	Y	10

Q1—Was true randomization used for assignment of participants to treatment groups? Q2—Was allocation to treatment groups concealed? Q3—Were treatment groups similar at the baseline? Q4—Were participants blind to treatment assignment? Q5—Were those delivering treatment blind to treatment assignment? Q6—Were outcomes assessors blind to treatment assignment? Q7—Were treatment groups treated identically other than the intervention of interest? Q8—Was follow-up complete and if not, were differences between groups adequately described and analyzed? Q9—Were participants analyzed in the groups to which they were randomized? Q10—Were outcomes measured in the same way for treatment groups? Q11—Were outcomes measured in a reliable way? Q12—Was appropriate statistical analysis used? Q13—Was the trial design appropriate and any deviations from standard RCT design accounted for in analysis? Abbreviations: Y = yes; N = no; U = unclear. * Total score represents the sum of “yes” responses across the 13 domains of the JBI Critical Appraisal Checklist for RCTs [12].

## Data Availability

The data supporting the findings of this study are available within the article and Supplementary Materials.

## Supplementary Materials

The following supporting information can be downloaded at https://www.mdpi.com/article/10.3390/nu18142258/s1. Supplementary Table S1: Search strategies for all databases; Supplementary Table S2: Detailed lifestyle intervention characteristics of the included studies; Supplementary Figure S1: Leave-one-out sensitivity analysis for gestational weight gain.
