# Supplementary material for: Effect of Lifestyle Interventions on Gestational Weight Gain Among Pregnant Women with Overweight: A Systematic Review and Meta-Analysis of Randomized Controlled Trials
Source: Nutrients. 2026 Jul 10;18(14):2258. doi: 10.3390/nu18142258 (PMC13414732; doi:10.3390/nu18142258)
Supplement: Supplementary file 1 [file nutrients-18-02258-s001.zip › nutrients-4392518-supplementary.pdf]

Supplementary Materials

Table S1. Search strategies for all databases.

Search strategy for PubMed (2015-2025).

PICO	Search Terms	Number of studies
Population	1. pregnan* OR gestation* OR "pregnant wom?n" OR "expectant mother*" OR maternal OR "pre-pregnancy" OR prepregnancy OR antenatal OR prenatal OR perinatal OR "antenatal care" OR "prenatal care" OR "Pregnancy"[Mesh] OR "Pregnant People"[Mesh]	613,955
	2. overweight OR "body mass index" OR BMI OR "Overweight"[Mesh]	368,299
	3. 1 AND 2	35,957
Intervention	4. diet* OR nutrition* OR "diet therapy" OR "diet modification" OR "dietary modification" OR "diet restriction" OR "dietary restriction" OR "diet counseling" OR "dietary counseling" OR "diet consultation" OR "dietary consultation" OR "dietary management" OR "diet intervention" OR "dietary intervention" OR "nutritional counseling" OR "nutritional consultation" OR "Diet"[Mesh] OR "Diet Therapy"[Mesh] OR "Nutrition Therapy"[Mesh]	867,522
	5. exercise* OR "physical activity" OR "physical exertion" OR "motor activity" OR walk* OR step* OR pedometer OR "step count" OR run* OR jog* OR swim* OR cycl* OR bicycl* OR yoga OR sport* OR "physical training" OR "strength training" OR "resistance training" OR "aerobic training" OR "endurance training" OR "Exercise"[Mesh] OR "Exercise Therapy"[Mesh] OR "Physical Exertion"[Mesh]	1,959,784

PICO	Search Terms	Number of studies
	6. lifestyle* OR "lifestyle intervention*" OR "life style" OR "lifestyle management*" OR "lifestyle modification*" OR education* OR counsel* OR consult* OR advice* OR booklet* OR "health education" OR "health promotion" OR behavior* OR behaviour* OR "behavior change" OR "behaviour change" OR "behavioral counseling" OR "behavioural counselling" OR "Life Style"[Mesh] OR "Health Behavior"[Mesh] OR "Health Promotion"[Mesh] OR "Health Education"[Mesh]	3,168,625
	7. 4 OR 5 OR 6	5,112,010
outcome	8. "gestational weight gain" OR GWG OR "pregnancy weight gain" OR "maternal weight gain" OR "weight gain" OR "weight change" OR "weight control" OR "Gestational Weight Gain"[Mesh]	54,406
	9. 3 AND 7 AND 8	3,672
	10. Filters: Randomized Controlled Trial	337

Search strategy for Embase (2015–2025)

PICO	Search Terms	Number of studies
Population	1. (pregnan*:ti,ab,kw OR gestation*:ti,ab,kw OR "pregnant wom?n":ti,ab,kw OR "expectant mother*":ti,ab,kw OR maternal:ti,ab,kw OR "pre-pregnancy":ti,ab,kw OR prepregnancy:ti,ab,kw OR antenatal:ti,ab,kw OR prenatal:ti,ab,kw OR perinatal:ti,ab,kw OR "antenatal care":ti,ab,kw OR "prenatal care":ti,ab,kw OR 'pregnancy'/exp) AND [2015-2025]/py	726,812
	2. (overweight:ti,ab,kw OR "body mass index":ti,ab,kw OR bmi:ti,ab,kw OR 'overweight'/exp) AND [2015-2025]/py	816,502
	3. 1 AND 2	68,274
Intervention	4. (diet*:ti,ab,kw OR nutrition*:ti,ab,kw OR "diet therapy":ti,ab,kw OR "diet modification":ti,ab,kw OR "dietary modification":ti,ab,kw OR "diet restriction":ti,ab,kw OR "dietary restriction":ti,ab,kw OR "diet counseling":ti,ab,kw OR "dietary counseling":ti,ab,kw OR "diet consultation":ti,ab,kw OR "dietary consultation":ti,ab,kw OR "dietary management":ti,ab,kw OR "diet intervention":ti,ab,kw OR "dietary intervention":ti,ab,kw OR "nutritional counseling":ti,ab,kw OR "nutritional consultation":ti,ab,kw OR 'diet'/exp OR 'diet therapy'/exp OR 'nutrition therapy'/exp) AND [2015-2025]/py	893,437
	5. (exercise*:ti,ab,kw OR "physical activity":ti,ab,kw OR "physical exertion":ti,ab,kw OR "motor activity":ti,ab,kw OR walk*:ti,ab,kw OR step*:ti,ab,kw OR pedometer:ti,ab,kw OR "step count":ti,ab,kw OR run*:ti,ab,kw OR jog*:ti,ab,kw OR swim*:ti,ab,kw OR cycl*:ti,ab,kw OR bicycl*:ti,ab,kw OR yoga:ti,ab,kw OR sport*:ti,ab,kw OR "physical training":ti,ab,kw OR "strength training":ti,ab,kw OR "resistance training":ti,ab,kw OR "aerobic training":ti,ab,kw OR	2,346,199

PICO	Search Terms	Number of studies
	"endurance training":ti,ab,kw OR 'exercise'/exp OR 'exercise therapy'/exp OR 'physical exertion'/exp) AND [2015-2025]/py	
	6. (lifestyle*:ti,ab,kw OR "lifestyle intervention*":ti,ab,kw OR "life style":ti,ab,kw OR "lifestyle management*":ti,ab,kw OR "lifestyle modification*":ti,ab,kw OR education*:ti,ab,kw OR counsel*:ti,ab,kw OR consult*:ti,ab,kw OR advice*:ti,ab,kw OR booklet*:ti,ab,kw OR "health education":ti,ab,kw OR "health promotion":ti,ab,kw OR behavior*:ti,ab,kw OR behaviour*:ti,ab,kw OR "behavior change":ti,ab,kw OR "behaviour change":ti,ab,kw OR "behavioral counseling":ti,ab,kw OR "behavioural counselling":ti,ab,kw OR 'life style'/exp OR 'health behavior'/exp OR 'health promotion'/exp OR 'health education'/exp) AND [2015-2025]/py	2,468,408
	7. 4 OR 5 OR 6	4,998,240
outcome	8. ("gestational weight gain":ti,ab,kw OR gwg:ti,ab,kw OR "pregnancy weight gain":ti,ab,kw OR "maternal weight gain":ti,ab,kw OR "weight gain":ti,ab,kw OR "weight change":ti,ab,kw OR "weight control":ti,ab,kw OR 'gestational weight gain'/exp) AND [2015-2025]/py	79,073
	9. 3 AND 7 AND 8	4,976
	10. 9 AND 'randomized controlled trial'/de AND 'article'/it	343

Search strategy for Scopus (2015–2025)

PICO	Search Terms	Number of studies
Population	1. TITLE-ABS-KEY (pregnan* OR gestation* OR "pregnant wom?n" OR "expectant mother*" OR maternal OR "pre-pregnancy" OR prepregnancy OR antenatal OR prenatal OR perinatal OR "antenatal care" OR "prenatal care") AND PUBYEAR > 2014 AND PUBYEAR < 2026	700,481
	2. TITLE-ABS-KEY (overweight OR "body mass index" OR BMI) AND PUBYEAR > 2014 AND PUBYEAR < 2026	339,578
	3. 1 AND 2	32,610
Intervention	4. TITLE-ABS-KEY (diet* OR nutrition* OR "diet therapy" OR "diet modification" OR "dietary modification" OR "diet restriction" OR "dietary restriction" OR "diet counseling" OR "dietary counseling" OR "diet consultation" OR "dietary consultation" OR "dietary management" OR "diet intervention" OR "dietary intervention" OR "nutritional counseling" OR "nutritional consultation") AND PUBYEAR > 2014 AND PUBYEAR < 2026	1,060,078
	5. TITLE-ABS-KEY (exercise* OR "physical activity" OR "physical exertion" OR "motor activity" OR walk* OR step* OR pedometer OR "step count" OR run* OR jog* OR swim* OR cycl* OR bicycl* OR yoga OR sport* OR "physical training" OR "strength training" OR "resistance training" OR "aerobic training" OR "endurance training") AND PUBYEAR > 2014 AND PUBYEAR < 2026	5,323,025
	6. TITLE-ABS-KEY (lifestyle* OR "lifestyle intervention*" OR "life style" OR "lifestyle management*" OR "lifestyle modification*" OR education* OR counsel* OR consult* OR advice* OR booklet* OR "health education" OR "health promotion" OR behavior* OR	5,663,867

PICO	Search Terms	Number of studies
	behaviour* OR "behavior change" OR "behaviour change" OR "behavioral counseling" OR "behavioural counselling") AND PUBYEAR > 2014 AND PUBYEAR < 2026	
	7. 4 OR 5 OR 6	10,860,965
Outcome	8. TITLE-ABS-KEY ("gestational weight gain" OR GWG OR "pregnancy weight gain" OR "maternal weight gain" OR "weight gain" OR "weight change" OR "weight control") AND PUBYEAR > 2014 AND PUBYEAR < 2026	119,216
Study design	9. TITLE-ABS-KEY (random* OR "randomized controlled trial" OR "randomised controlled trial" OR "controlled clinical trial" OR rct) AND PUBYEAR > 2014 AND PUBYEAR < 2026	2,089,652
	10. 3 AND 7 AND 8 AND 9	499

Search strategy for CINAHL (via EBSCOhost) (2015–2025)

PICO	Search Terms	Number of studies
Population	1. (MH "Pregnancy+" OR MH "Prenatal Care" OR TI pregnan* OR AB pregnan* OR TI gestation* OR AB gestation* OR TI "pregnant wom?n" OR AB "pregnant wom?n" OR TI "expectant mother*" OR AB "expectant mother*" OR TI maternal OR AB maternal OR TI antenatal OR AB antenatal OR TI prenatal OR AB prenatal OR TI perinatal OR AB perinatal OR TI prepregnancy OR AB prepregnancy OR TI "pre-pregnancy" OR AB "pre-pregnancy" OR TI "antenatal care" OR AB "antenatal care" OR TI "prenatal care" OR AB "prenatal care")	92,329
	2. (MH "Body Mass Index" OR MH "Overweight+" OR TI overweight OR AB overweight OR TI "body mass index" OR AB "body mass index" OR TI BMI OR AB BMI)	55,520
	3. 1 AND 2	5,633
Intervention	4. (MH "Diet Therapy+" OR MH "Nutrition Therapy+" OR TI diet* OR AB diet* OR TI nutrition* OR AB nutrition* OR TI "diet therapy" OR AB "diet therapy" OR TI "diet modification" OR AB "diet modification" OR TI "dietary modification" OR AB "dietary modification" OR TI "diet restriction" OR AB "diet restriction" OR TI "dietary restriction" OR AB "dietary restriction" OR TI "diet counseling" OR AB "diet counseling" OR TI "dietary counseling" OR AB "dietary counseling" OR TI "diet consultation" OR AB "diet consultation" OR TI "dietary consultation" OR AB "dietary consultation" OR TI "dietary management" OR AB "dietary management" OR TI "diet intervention" OR AB "diet intervention" OR TI "dietary intervention" OR AB "dietary intervention" OR TI "nutritional counseling" OR AB "nutritional counseling" OR TI "nutritional consultation" OR AB "nutritional consultation")	88,647

PICO	Search Terms	Number of studies
	5. (MH "Exercise+" OR MH "Motor Activity+" OR MH "Physical Fitness+" OR TI exercise* OR AB exercise* OR TI "physical activity" OR AB "physical activity" OR TI "physical exertion" OR AB "physical exertion" OR TI "motor activity" OR AB "motor activity" OR TI walk* OR AB walk* OR TI step* OR AB step* OR TI pedometer OR AB pedometer OR TI "step count" OR AB "step count" OR TI run* OR AB run* OR TI jog* OR AB jog* OR TI swim* OR AB swim* OR TI cycl* OR AB cycl* OR TI bicycl* OR AB bicycl* OR TI yoga OR AB yoga OR TI sport* OR AB sport* OR TI "physical training" OR AB "physical training" OR TI "strength training" OR AB "strength training" OR TI "resistance training" OR AB "resistance training" OR TI "aerobic training" OR AB "aerobic training" OR TI "endurance training" OR AB "endurance training")	162,246
	6. (MH "Life Style+" OR MH "Health Education+" OR MH "Health Promotion+" OR TI lifestyle* OR AB lifestyle* OR TI "lifestyle intervention*" OR AB "lifestyle intervention*" OR TI "life style" OR AB "life style" OR TI "lifestyle management*" OR AB "lifestyle management*" OR TI "lifestyle modification*" OR AB "lifestyle modification*" OR TI education* OR AB education* OR TI counsel* OR AB counsel* OR TI consult* OR AB consult* OR TI advice* OR AB advice* OR TI booklet* OR AB booklet* OR TI "health education" OR AB "health education" OR TI "health promotion" OR AB "health promotion" OR TI behavior* OR AB behavior* OR TI behaviour* OR AB behaviour* OR TI "behavior change" OR AB "behavior change" OR TI "behaviour change" OR AB "behaviour change" OR TI "behavioral counseling" OR AB "behavioral counseling" OR TI "behavioural counselling" OR AB "behavioural counselling")	312,345

PICO	Search Terms	Number of studies
	7. 4 OR 5 OR 6	486,776
Outcome	8. (MH "Weight Gain+" OR MH "Body Weight Changes+" OR TI "gestational weight gain" OR AB "gestational weight gain" OR TI GWG OR AB GWG OR TI "pregnancy weight gain" OR AB "pregnancy weight gain" OR TI "maternal weight gain" OR AB "maternal weight gain" OR TI "weight gain" OR AB "weight gain" OR TI "weight change" OR AB "weight change" OR TI "weight control" OR AB "weight control")	40,817
Study design	9. (MH "Randomized Controlled Trials+" OR MH "Clinical Trials+" OR TI random* OR AB random* OR TI "randomized controlled trial" OR AB "randomized controlled trial" OR TI "randomised controlled trial" OR AB "randomised controlled trial" OR TI "controlled clinical trial" OR AB "controlled clinical trial" OR TI rct OR AB rct)	180,047
	10. 3 AND 7 AND 8 AND 9	298

Table S2. Detailed lifestyle intervention characteristics of the included studies

	Author (year)	Materials	Intervention provider(s)	Location
1	Peccei (2017) [14]	Brochure, booklet, pedometer, meal plans	Dietitian	Clinic
2	Sagedal (2017) [15]	Booklets, website, cooking class	Dietitian; Public health personnel; Physical therapist; Exercise specialist	Clinic and local gyms
3	Gallagher (2018) [16]	Diabetes Prevention Program and Look AHEAD curricula	Nutritionist; Counselor	Clinic
4	Van Horn (2018) [17]	DASH materials, mobile applications, pedometers, digital media, e-mails, website	Dietitian; Lactation consultant	Clinic and home
5	Buckingham-Schutt (2019) [18]	Wearable activity tracker, meal plans, weight-gain chart	Dietitian	Clinic and home
6	Kunath (2019) [19]	Educational materials, weight-gain charts, PA brochures, pedometers	Midwife; Obstetrician/Gynecologist; Medical assistant	Clinic
7	Ferrara (2020) [20]	Weight-gain charts and workbook	Dietitians	Clinic
8	Hajian (2020) [21]	Educational booklets and pamphlets	Researcher; Nutritionist	Clinic
9	Liu (2021) [22]	Study handouts, weight-gain charts, pedometers, weight scale, digital media, online support group	Intervention staff	Clinic and home
10	Atkinson (2022) [23]	Counselling materials, recipes, meal plans, low-fat dairy foods, pedometer, accelerometer, Health Canada educational materials	Nutritionist; Exercise physiologist; Research assistant	Clinic

	Author (year)	Materials	Intervention provider(s)	Location
11	Krebs (2022) [24]	Websites, mobile applications, SMS reminder, goal-setting template, and counseling guidance tools	Obstetrician/Gynecologist; Midwife	Clinic
12	Sartorelli (2023) [25]	Educational folders with key messages and illustrative images	Nutritionist	Clinic
13	Chen (2023 [26]; 2024 [27])	Mobile application, wearable activity tracker, SMS reminders	Nurse; Healthcare professional	Clinic
14	Cabre (2025) [28]; Kebbe (2025) [29]	Weight scale, wearable activity tracker, exercise equipment, meal preparation tools, educational media, online support group	Health coach; Research assistant	Clinic and home
15	Su (2025) [30]	Mobile application with self-monitoring, educational, reminder, support, and reward features	Multidisciplinary team	Clinic and home

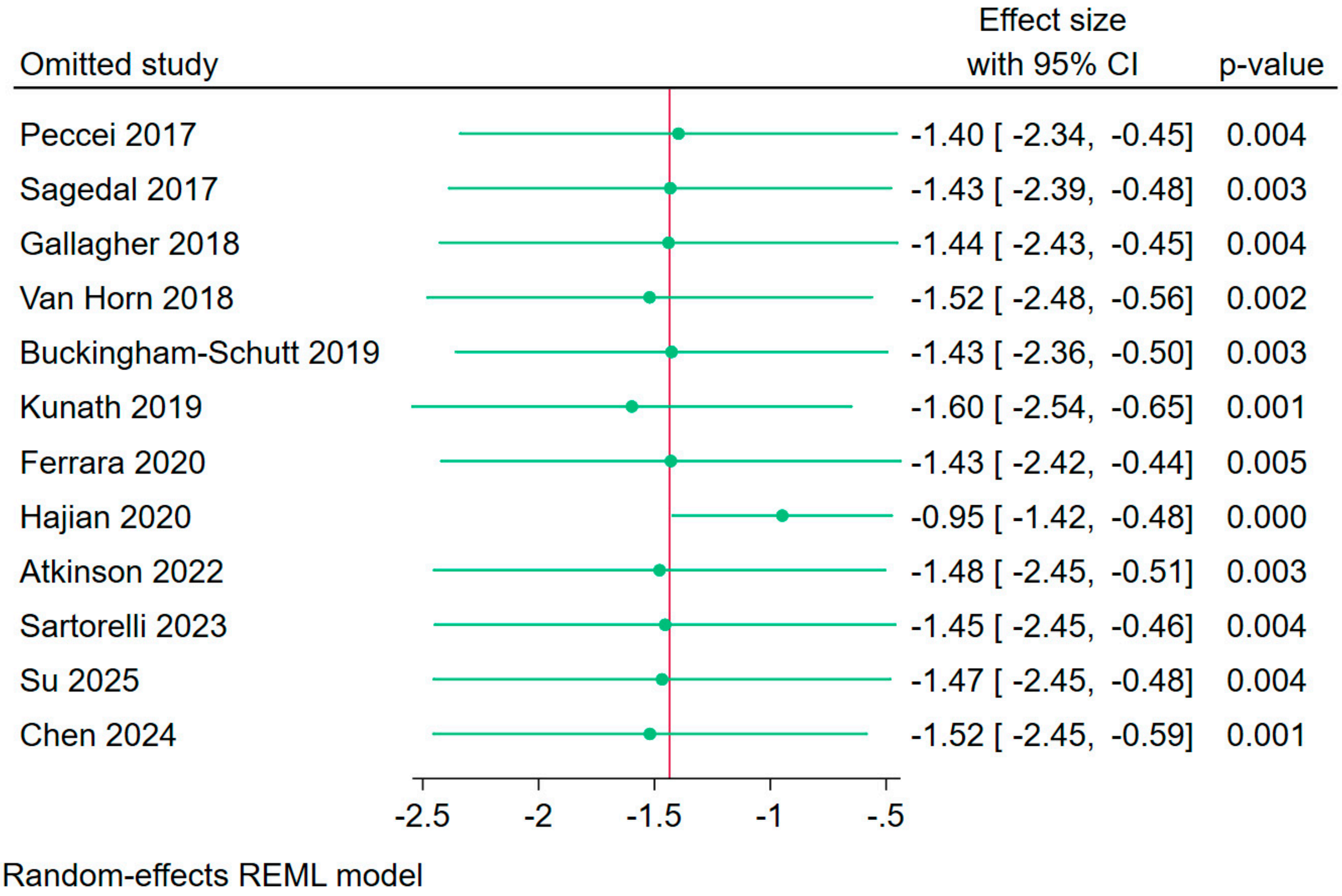

Figure S1. Leave-one-out sensitivity analysis for gestational weight gain.

Sequential omission of individual studies showed that the pooled effect estimate remained statistically significant and consistently favored the intervention group.
